# Development and validation of a computer program for histoanatomical morphometric analysis of the bowel wall in children with Hirschsprung’s disease

**DOI:** 10.1186/s13000-026-01779-7

**Published:** 2026-03-21

**Authors:** Tebin Hawez, Tobias Erlöv, Christina Granéli, Gustav Andersson, Tyra Lundberg, Emma Fransson, Maria Evertsson, Magnus Cinthio, Pernilla Stenström

**Affiliations:** 1 Department of Clinical Sciences Lund/Pediatrics, Lund University, Lund, Sweden; 2 Department of Pediatric Surgery, Children’s Hospital, Skåne University Hospital, Lund, Sweden; 3 Department of Biomedical Engineering, The Faculty of Engineering, Lund University, Lund, Sweden; 4 Department of Clinical Genetics, Pathology and Molecular Diagnostics, Skåne University Hospital, Lund, Sweden; 5 Department of Clinical Sciences Lund/Biomedical Engineering, Lund University, Lund, Sweden

**Keywords:** Bowel wall, Computer program, Histopathology, Histoanatomical morphometrics

## Abstract

**Background:**

Developing new diagnostic imaging methods that provide detailed visualization of the histoanatomy of tissues and organs requires precise histomorphometric evaluation. With a particular focus on bowel, this study aimed to develop and validate a computer program for reliable and efficient assessment of bowel wall histomorphometry.

**Methods:**

A MATLAB-based computer program was developed in-house to manually delineate and automatically calculate mean layer thickness of the muscularis propria layers, submucosa and mucosa in histopathology images of bowel wall specimens from patients operated on for Hirschsprung’s disease. Validation included assessment of inter- and intra-observer reliability and agreement for generated mean thicknesses, as well as comparison with manual measurements (mean of 10 thickness measurement points). Reliability was assessed through intraclass correlation coefficient (ICC) (good > 0.75) and agreement through Bland-Altman analysis (good= mean close to zero and spread within 2 standard deviations).

**Results:**

The program allowed for import of histopathology images for bowel wall layer analysis. After manual layer delineation, the program automatically calculated mean layer thickness (mm) ± standard deviation based on approximately 3000 measurement points. For the inter-observer analyses the reliability was moderate to good in the majority of the histoanatomic layers (ICC range 0.6–0.9) and agreement was similarly good based on the Bland-Altman analyses. Also, the intra-observer reliability ranged between good and excellent in the majority of histoanatomic layers (ICC range 0.7-1.0). The high-precision layer delineation and mean thickness extraction per image took 15 min, compared to 60 min for manual measurement.

**Conclusions:**

The developed computer program enables precise and time-efficient measurements of histoanatomic layer thicknesses in histopathology images of bowel wall, with good reliabilities and agreements between examinators. The program’s application is useful in histomorphometric evaluations for advancing diagnostic imaging techniques.

**Supplementary Information:**

The online version contains supplementary material available at 10.1186/s13000-026-01779-7.

## Background

 Currently, new diagnostic imaging methods are being developed to enable detailed histoanatomical visualization of tissues and organs based on histomorphometric analyses [1–4]. To support the development and validation of these imaging technologies - particularly those focused on the bowel wall and diagnostics in Hirschsprung’s disease [5–7], there is an increasing need for more precise histomorphometric anatomical reference data, as these structures are directly reflected in high-resolution imaging. Since performing manual measurements in histopathological images, e.g. of bowel wall, is time-consuming, and only a limited number of measurement points can be assessed, a high-precision and efficient measurement method is sought. Such a method could also help reduce observer bias and internal variability, and may enable histomorphometric assessment of other organs. For that purpose, a computer program facilitating assessment of bowel wall histomorphometry was warranted.

The primary aim was to develop a computer program with a user-friendly user interface capable of performing reliable, efficient and reproducible high-precision histoanatomical morphometric measurements of bowel wall in histopathology images of bowel wall specimens resected from children undergoing surgery for Hirschsprung’s disease. The secondary aim was to validate this computer program between users (inter-observer analyses) and between different assessments by the same user (intra-observer analyses). Additionally, the aim was to validate the computer program against manual measurements.

## Materials and methods

### Setting and patients

This was a translational development and validation study, conducted using histopathological images of bowel wall specimens from patients operated on for Hirschsprung’s disease at a Department of Pediatric Surgery serving as a national referral center for Hirschsprung’s disease. The study was part of a larger translational project involving pediatric surgeons, biomedical engineers, and pathologists, aiming to develop a real time diagnostic tool for diagnostics of Hirschsprung’s disease by ultra-high frequency ultrasound [4–8].

All patients diagnosed with Hirschsprung’s disease who underwent surgical resection of aganglionic segments between April 2018, and January 2024 at the department were eligible for inclusion. Inclusion criteria were diagnosis of rectosigmoid Hirschsprung’s disease with a maximum aganglionic segment length of 25 cm, age below 1 year, and body weight not exceeding 10 kilos at the time of surgery. The selection criteria were chosen to ensure a homogeneous patient group, thereby supporting the development of a computer program tailored to the most common variant of Hirschsprung’s disease.

### Tissue samples and specimen treatment

Following surgical resection of the diseased aganglionic segment, the resected bowel specimen was pinned to a cork mat, formalin-fixed and submitted to the Department of Clinical Genetics and Pathology for histopathological analysis. The resected specimen was cross-sectioned along its length, and sections from all diagnostically relevant levels were paraffin-embedded and stained with haematoxylin-eosin and immunohistochemistry according to local protocols. Microscopic imaging was used to assess aganglionic and ganglionic segments. All histopathological images were archived in the histopathology image management software Sectra PACS IDS7 (Sectra AB, Linköping, Sweden). For each patient, paired high-quality images of aganglionic and ganglionic tissue were selected and saved. The selection criteria were images with minimal artefacts or damage and with clearly distinguishable histoanatomical structures.

### Computer program

A custom computer program was developed in MATLAB version 9.13 (R2022b, The MathWorks Inc., Natick, MA, USA). The development team included biomedical engineers, pathologists, and pediatric surgeons, with experience from similar software development for ultra-high frequency ultrasound of bowel wall [9]. The histoanatomy computer program is a semi-automatic method designed to (a) enable accurate and flexible manual delineation, (b) perform automatic high-density calculations of bowel wall layer thicknesses, (c) offer a user-friendly interface, and (d) allow assessment of colour densities within each histoanatomical layer. The last requirement was based on the theory that more collagen-dense tissue would exhibit more intense staining. This is to be investigated and validated in a future study.

The program development was guided by the translational team and users (clinicians). During the development, the implemented technical refinements were tested continuously. Iterative updates were based on user feedback from simulated image assessments. Optimization of the interface and functionality was evaluated regularly against the predefined criteria. A compiled version of the program was finalized to ensure consistency and prevent code modification.

### Validation methodology

For each histopathological image of aganglionic and ganglionic bowel, the following bowel wall layers were measured using the computer program: muscularis propria externa (longitudinal muscle, hereafter referred to as muscularis externa), the myenteric tissue layer (situated between the longitudinal and circular muscularis propria layers), muscularis propria interna (circular muscle, hereafter referred to as muscularis interna), submucosa, and mucosa. The full bowel wall thickness was calculated from these parameters. Histopathology images of the transition zone were not included in this study.

Validation of the computer program included analyses of inter- and intra-observer reliability and agreement of histoanatomic layer thickness measurements, as well as similar analyses between manual and computer program measurements. Inter-observer reliability and agreement were assessed between two independent observers. Images were organized into two separate folders: one containing aganglionic bowel samples and the other containing ganglionic samples. Within each folder, the images were blinded to ensure unbiased assessment. Each observer performed the analysis independently, without access to the other’s evaluation.

Intra-observer reliability and agreement was assessed based on two separate evaluations of images by a single observer. The observer did not have access to the initial measurements, to avoid biased re-assessment.

The semi-automatic assessment by the computer program was validated by analysing reliability and agreement versus manual assessment. Manual assessment of bowel wall layers, using caliper tools in the conventional histopathology image management software Sectra PACS ID7 (Sectra AB, Linköping, Sweden) has been published previously [6]. The thicknesses in the manual assessment were calculated as the mean layer thickness of 10 distinct points per sample, selected by dividing the circumference into 10 equally distributed segments. The decision to use ten manual measurements was justified by a descriptive and approximative calculation showing that this number provided a representative sampling of layer thickness values, while avoiding an excessive measurement burden given the time-consuming nature of the manual procedure. The manual and automated calculations were treated as two distinct measurement approaches for comparison, and neither was regarded as ground truth.

### Statistics

Reliability was evaluated using the intraclass correlation coefficient (ICC) which was based on a two-way mixed-effects model, absolute agreement, and single observers [10–12]. ICC values were interpreted according to the definitions [10]: less than 0.5 indicated poor reliability, between 0.5 and 0.75 indicated moderate reliability, between 0.75 and 0.9 indicated good reliability, and values greater than 0.9 indicated excellent reliability. Agreement was assessed using Bland-Altman plots [12–14]. In Bland-Altman analysis, the acceptable degree of agreement is typically determined by any clinical relevance. Therefore, the agreement was considered acceptable based on an even distribution of individual differences implying a mean difference close to zero and the majority of datapoints falling within ± two standard deviations.

Data management and statistical analysis were conducted using Microsoft Excel and IBM SPSS statistics version 27. Bland-Altman plots were generated using R (version 4.3.1; R Core Team, 2023). Statistical support was provided by statisticians at the government-funded Clinical Studies Sweden – Forum South.

In the main results presentation and visualization, analyses of the muscularis interna and externa were selected, as these layers have been identified as the most relevant for distinguishing between aganglionosis and ganglionosis [4, 6, 7]. A complete overview of all analyses (inter- and intra-observer, and manual vs. semi-automatic) across all histoanatomic layers is provided in the supplementary material.

### Development of functionality and technology of the semi-automatic computer program

**Fig. 1:**
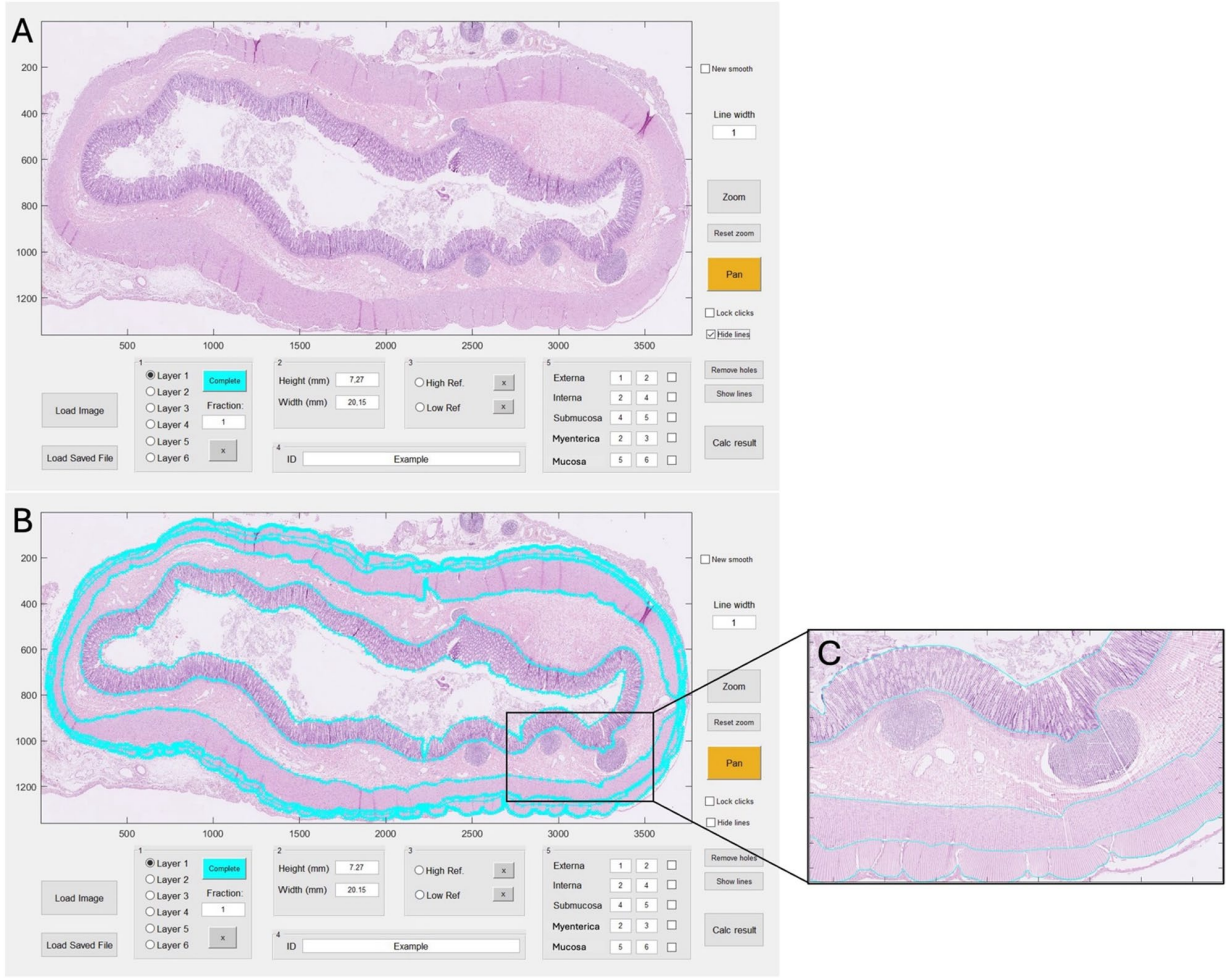
User interface of the in-house developed computer program for semi-automatic histomorphometric bowel wall assessment After importing an image to the software (**A**), the user can delineate the inner and outer borders of the layers muscularis externa, the myenteric tissue layer between the muscularis externa and interna, muscularis interna, submucosa, and mucosa by clicking in the image. The delineated layer borders are shown as blue lines (**B**). The program calculates layer thicknesses automatically by measuring the shortest perpendicular distance between the inner and outer layer boundaries at measurement intervals of 14 μm, resulting in thousands of thickness measurements per layer (**C**)

**Fig. 2:**
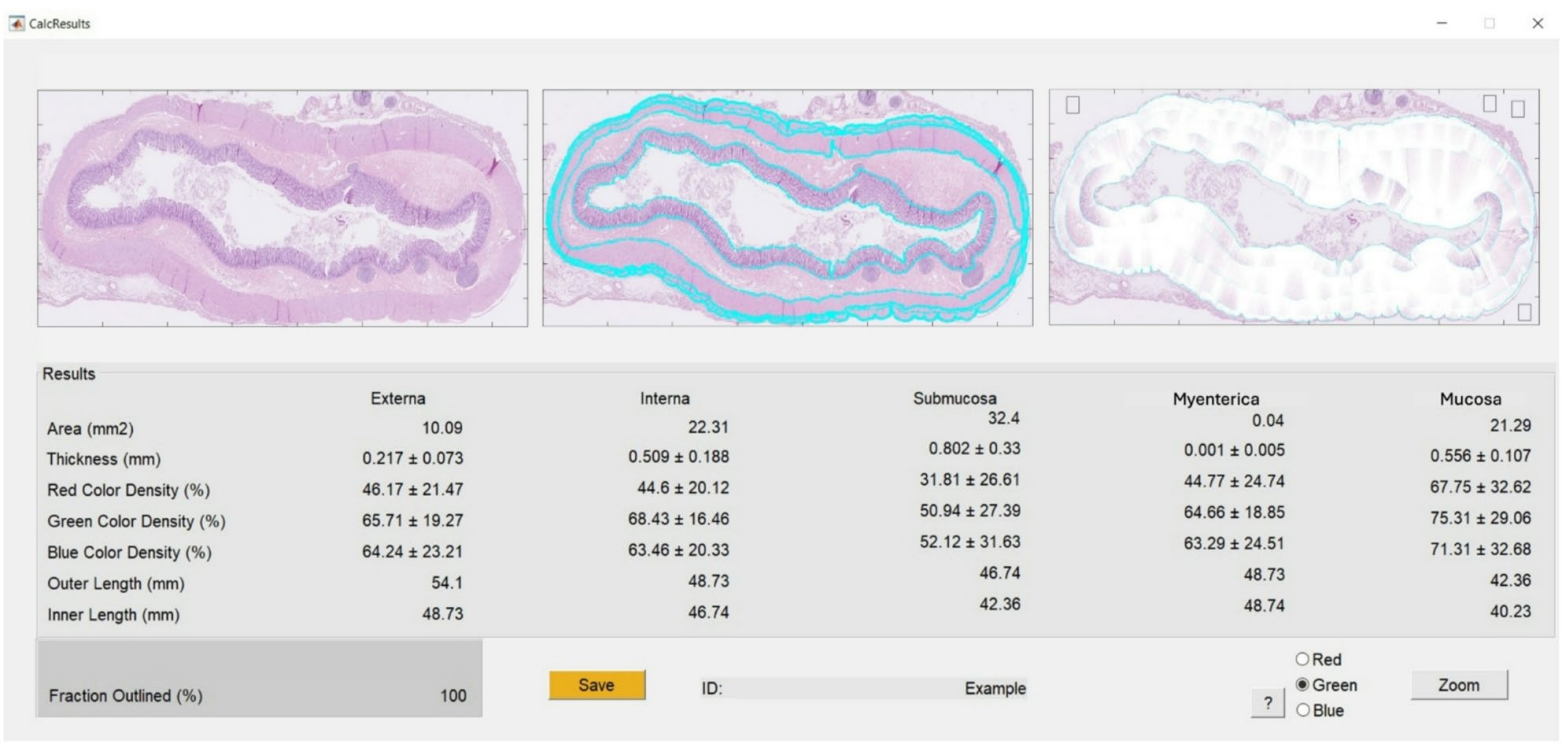
The results window of the in-house developed computer program for semi-automatic histomorphometric bowel wall assessment After delineation of inner and outer borders of the histoanatomic layers, automatic calculation is undertaken, resulting in mean layer thicknesses, layer areas, lengths of inner and outer layer boundaries and layer colour densities in the areas

The development process included several iterative phases aimed at enhancing the precision and flexibility of manual delineation and optimizing the output and visualization of the automated calculations. The final product was a computer program that allowed for import of histopathological images for bowel layer analysis of muscularis externa, the myenteric tissue layer, muscularis interna, submucosa and mucosa as shown in Fig. 1. The delineation was ultimately designed to allow manual marking of the inner and outer borders of each specific layer in the histopathology images. The position of each marking in the delineation, and space between these markings, was determined by the user. The marking process allowed easy adjustments of the marking positions, in order to accurately follow the borders of the histoanatomic layers. The software was programmed to automatically interpolate between the markings to generate a detailed, continuous line along the inner and outer borders of each layer (Fig. 1B). Once the delineations of both the inner and outer boundaries for a specific layer were determined to be accurate by the user, the program automatically calculated the layer thickness. This was calculated by the shortest perpendicular distance (or near perpendicular) between the inner and outer layer boundaries at pixel level (measurement intervals of 14 μm in this study). An additional function was incorporated, allowing the direction of the line across the layer, i.e. the angle of thickness measurement, to change smoothly along the layer. These features resulted in approximately 3000 measurements per layer (Fig. 1C). Based on this information the software was programmed to automatically calculate a mean layer thickness (mm) ± standard deviation, layer area (mm^2^), length of the inner and outer layer boundaries (mm), and a colour density (blue, red and green) for each layer. The total result of all automatic calculations was presented by the computer program in one results window (Fig. 2).

## Results

### Inter-observer reliability and agreement

**Fig. 3:**
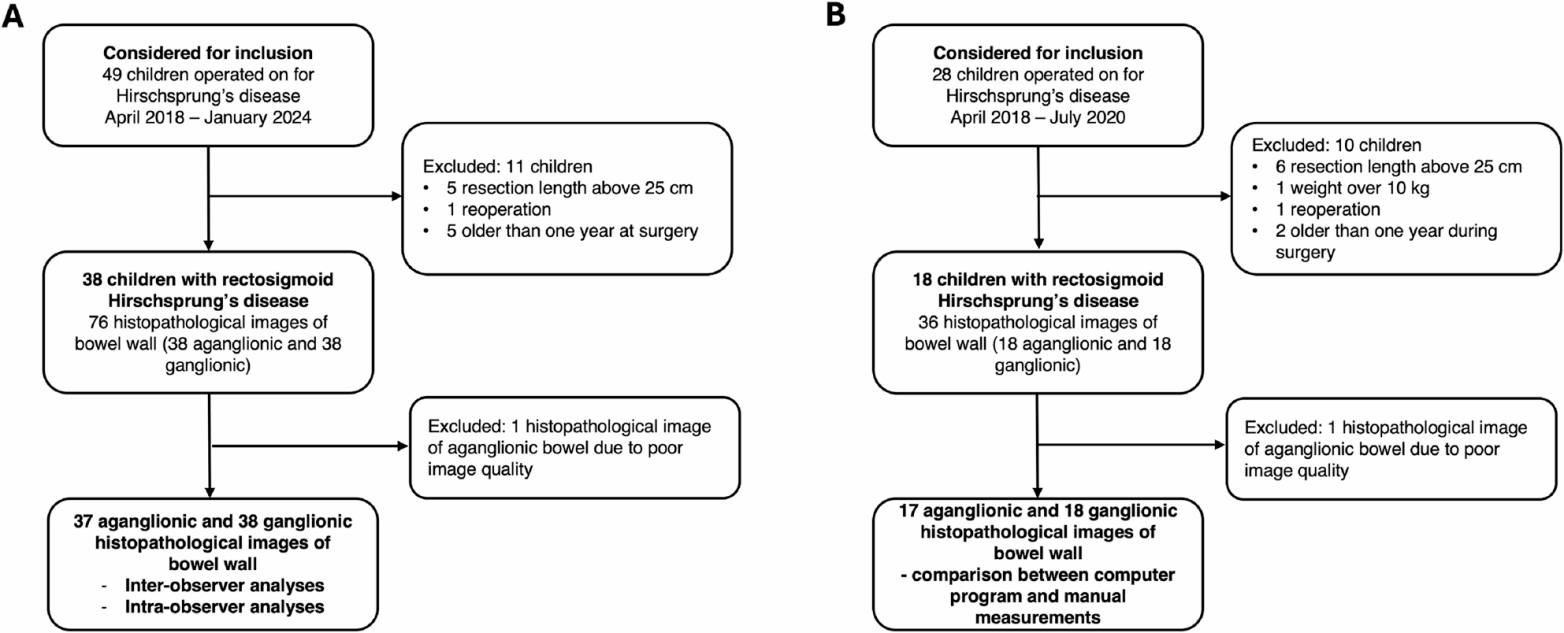
Flowchart of patients and images included **A** Inclusion criteria for the inter- and intra-observer validation. **B** Inclusion criteria for comparison with manual histomorphometric measurements

During the study period, 49 children underwent surgery for Hirschsprung’s disease. Based on the study inclusion criteria, 38 patients were included, from whom histopathology images of both aganglionic and ganglionic bowel wall was obtained. One aganglionic image was excluded due to poor image quality. In total, 37 aganglionic and 38 ganglionic histopathology images were assessed in the computer program and included in the inter- and intra-observer reliability and agreement analyses (Fig. 3A).

The inter-observer reliability, measured by ICC, between two computer program users, was moderate to good with the majority of ICCs ranging between 0.6 and 0.9 for the muscularis layers in both aganglionic bowel wall and ganglionic bowel wall (Table 1a and 1b), and also for the other layers (Tables 1 and 2, Supplementary). The inter-observer reliability for the muscularis layers was good for both layers in aganglionic bowel (Table 1a), whereas in ganglionic bowel, reliability was good only for the muscularis interna (Table 1b). Assessing all histoanatomic layers, in aganglionic bowel, the highest inter-observer reliability was that of the muscularis externa (ICC = 0.881), while in ganglionic bowel the highest reliability was noted for the submucosa (ICC = 0.882). The lowest degree of inter-observer reliability was that of the myenteric tissue layer thicknesses in both aganglionic bowel (ICC = 0.264) and ganglionic bowel (ICC = 0.101), (Tables 1 and 2, Supplementary).

**Table 1:** a. Inter-observer reliability of a computer program for histomorphometric analysis. Comparison of aganglionic segments. b. Inter-observer reliability of a computer program for histomorphometric analysis. Comparison of ganglionic segments

a. Inter-observer reliability of a computer program for histomorphometric analysis. Comparison of aganglionic segments				
Histoanatomical layer	Thickness observer 1 Median (Range)	Thickness observer 2 Median (Range)	Absolute difference Median (Range)	ICC^1^
Muscularis externa (mm) n=37	0.316 (0.164 – 1.227)	0.318 (0.161 – 0.988)	0.024 (-0.159 – 0.451)	0.881
Muscularis interna (mm) n=37	0.419 (0.235 – 0.752)	0.416 (0.215 – 0.827)	-0.002 (-0.290 – 0.281)	0.811

b. Inter-observer reliability of a computer program for histomorphometric analysis. Comparison of ganglionic segments				
Histoanatomical layer	Thickness observer 1 Median (Range)	Thickness observer 2 Median (Range)	Absolute difference Median (Range)	ICC^1^
Muscularis externa (mm) n=38	0.326 (0.130 - 0.679)	0.306 (0.102 - 0.559)	0.017 (-0.208 - 0.466)	0.672
Muscularis interna (mm) n=38	0.620 (0.204 - 0.994)	0.625 (0.252 - 1.111)	-0.007 (-0.490 - 0.231)	0.853

^1^ Intraclass Correlation Coefficient (ICC) <0.5 poor reliability; ICC 0.5 - 0.75 moderate reliability; ICC 0.75 - 0.9 good reliability; ICC>0.9 excellent reliability

The table presents inter-observer reliability between two observers using the in-house developed computer program for assessment of bowel wall histomorphometry in histopathology images of resected bowel specimens in children with Hirschsprung’s disease

**Fig. 4:**
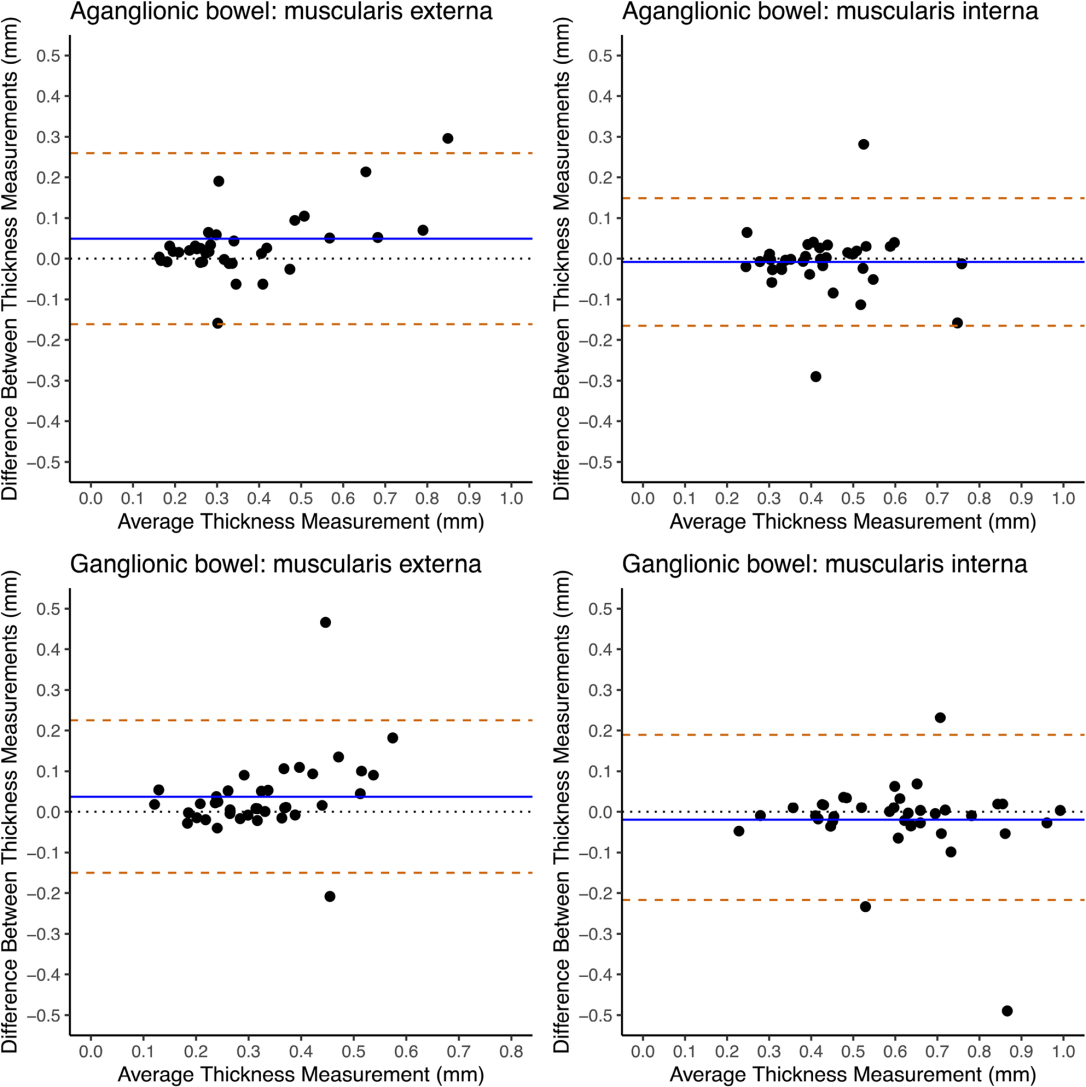
Bland-Altman plots visualizing inter-observer agreement of layer thickness measurements of the bowel wall Inter-observer agreement between two observers assessing bowel layer thickness measurements from histopathological images analysed in the in-house developed computer program for semi-automatic histomorphometric bowel wall assessment. The images were of aganglionic and ganglionic bowel wall from children operated on for Hirschsprung’s disease. The blue horizontal lines represent mean thickness difference. The yellow dashed horizontal lines indicate the limits of agreement, defined as the mean thickness difference ± two standard deviations of the differences

The inter-observer agreement was good (mean difference close to zero) for both aganglionic and ganglionic muscularis interna (difference − 0.008 mm and − 0.019 mm, respectively) (Fig. 4) and submucosa (difference − 0.012 mm and − 0.011 mm, respectively) (Fig. 1, Supplementary), as demonstrated by the Bland-Altman analyses. Despite a good agreement, still a few datapoints for both muscularis interna and submucosa fell outside ± two standard deviations. The lowest agreement in the Bland-Altman analyses was those of the myenteric tissue layer, generating a wide distribution of data points and a mean thickness difference of 0.014 mm in aganglionic bowel and 0.019 mm in ganglionic bowel (Fig. 1, Supplementary).

### Intra-observer reliability and agreement analyses

In intra-observer analyses (repeated assessments of one observer) the reliability of the computer program was considered good to excellent with ICCs for histoanatomic layers ranging from 0.7 to 1.0 in both aganglionic bowel and ganglionic bowel (Table 3, and 4, Supplementary). The strongest layer intra-observer ICCs were those for muscularis externa in aganglionic bowel (ICC = 0.969) and submucosa in ganglionic bowel (ICC = 0.983). The weakest intra-observer reliability was that for the mucosa in aganglionic bowel (ICC = 0.698) and for the myenteric tissue layer in ganglionic bowel (ICC = 0.418).

The agreement between intra-observer assessments was overall good as indicated by the Bland-Altman plots, with mean layer thickness differences close to zero and a narrow distribution of datapoints (Fig. 2, Supplementary). Compared with the inter-observer analyses, fewer data points fell outside the ± two standard deviations range. The highest agreements were noted for the muscularis interna, submucosa and mucosa in both aganglionosis and ganglionosis ranging in differences from – 0.014 to 0.004 mm (Fig. 2, Supplementary).

### Computer program vs. manual reliability and agreement

Manual assessment of histopathology images was performed between April 2018 and July 2020, during which 28 children underwent surgery for Hirschsprung’s disease. According to the inclusion criteria, 18 patients were included, from whom histopathology images of both aganglionic and ganglionic bowel wall were obtained. One aganglionic image was excluded due to poor image quality. In total, 17 aganglionic and 18 ganglionic histopathology images were included in the comparison between the computer program and manual assessments of layer thicknesses (Fig. 3B).

In reliability tests of the computer program versus manual measurements, good reliability was shown for muscularis externa (ICC = 0.883) and muscularis interna (ICC = 0.849) in aganglionic bowel (Table 2a), and muscularis interna (ICC = 0.766; Table 2b) and mucosa (ICC = 0.777; Table 6, Supplementary) in ganglionic bowel. The weakest reliability was that for the myenteric tissue layer shown by low ICCs in both aganglionosis (ICC = 0.164) and ganglionosis (ICC=-0.166) (Table 5, and 6, Supplementary).

**Table 2:** a. Reliability between manual measurements and a computer program for histomorphometric analysis. Comparison of aganglionic segments. b. Reliability between manual measurements and a computer program for histomorphometric analysis. Comparison of ganglionic segments

a. Reliability between manual measurements and a computer program for histomorphometric analysis. Comparison of aganglionic segments				
Histoanatomical layers	Thickness computer program measurements Median (Range)	Thickness manual measurements Median (Range)	Absolute difference Median (Range)	ICC^1^
Muscularis externa (mm) n = 17	0.412 (0.164–1.227)	0.451 (0.180–1.591)	-0.002 (-0.376–0.475)	0.883
Muscularis interna (mm) n = 17	0.461 (0.235–0.752)	0.445 (0.287–1.016)	0.002 (-0.264–0.092)	0.849

b. Reliability between manual measurements and a computer program for histomorphometric analysis. Comparison of ganglionic segments				
Histoanatomicallayers	Thickness computer program measurements Median (Range)	Thickness manual measurements Median (Range)	Absolute difference Median (Range)	ICC^1^
Muscularis externa (mm) n=18	0.364 (0.170 - 0.679)	0.284 (0.210 - 0.488)	0.022 (-0.081 - 0.451)	0.274
Muscularis interna (mm) n=18	0.683 (0.408 - 0.994)	0.619 (0.429 - 0.974)	-0.016 (-0.135 - 0.356)	0.766

^1^ Intraclass Correlation Coefficient (ICC) <0.5 poor reliability; ICC 0.5 - 0.75 moderate reliability; ICC 0.75 - 0.9 good reliability; ICC>0.9 excellent reliability

The table presents reliability between manual measurements and the in-house developed computer program for assessment of bowel wall histomorphometry in histopathology images of resected bowel specimens in children with Hirschsprung’s disease

**Fig. 5:**
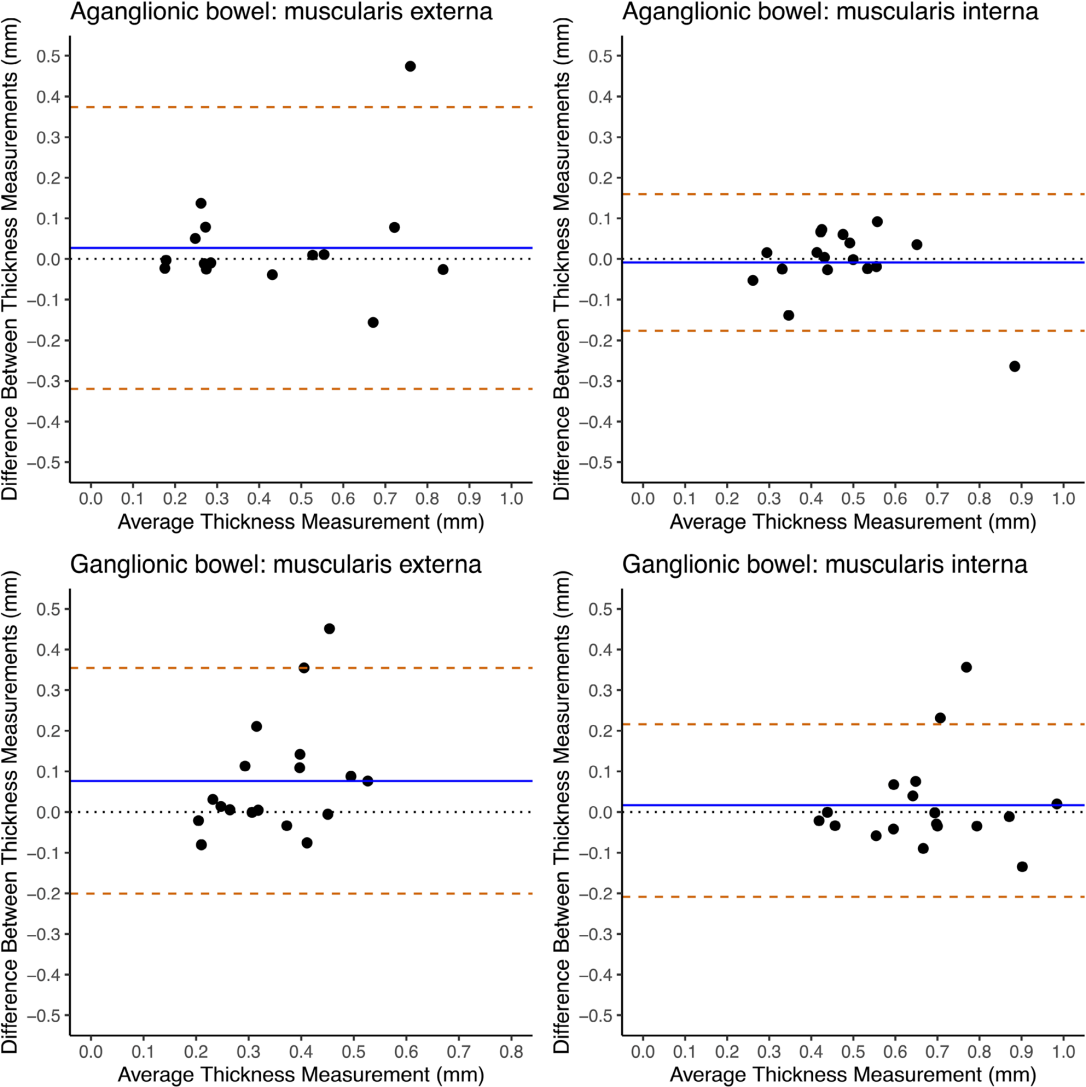
Bland-Altman plots visualizing agreement between manual and computer program measurements of bowel wall layer thicknesses Agreement between the in-house developed computer program for semi-automatic histomorphometric bowel wall assessment and manual assessment of layer thickness measurements from histopathological images. The images were of aganglionic and ganglionic bowel wall from children operated on for Hirschsprung’s disease. The blue horizontal lines represent mean thickness difference. The yellow dashed horizontal lines indicate the limits of agreement, defined as the mean thickness difference ± two standard deviations of the differences

The agreement between computer and manual assessments, as presented in Bland-Altman plots, varied largely between the histoanatomic layers. The highest agreement was that of the muscularis interna, with a generally narrow data point distribution and a mean difference of -0.009 mm in aganglionosis and 0.016 mm in ganglionosis, indicating a good agreement (Fig. 5). Low agreement in the Bland-Altman analysis was shown for the aganglionic and ganglionic submucosa (difference 0.198 mm and 0.201 mm, respectively) and myenteric tissue layer (difference − 0.005 mm and 0.010 mm, respectively), with both layers also presenting with a wide distribution of data points (Fig. 3, Supplementary). 

### Time efficacy

Layer delineation and thickness extraction for all layers in an image took 15 min using the computer program (approximately 3000 measurement points). In contrast, manual assessment based on 10 thickness measurement points in an image of bowel wall, including extraction of results, required 60 min.

## Discussion

This study describes the development and validation of a computer program designed for histomorphometric analysis of bowel wall in histopathology images. The final product determines mean histoanatomical layer thicknesses from thousands of point measurements per layer, presented within an intuitive and user-friendly interface. Validation tests showed both good reliability and strong agreement, both in interobserver analyses and across repeated measurements by the same observer in intra-observer analyses. These findings support the program’s potential to serve as a valid tool for histopathological assessment. The computer program was developed, evaluated and continuously refined by both designers and users, progressively meeting the predefined requirements. A key advantage of the computer program was its time efficiency, allowing completeness of all bowel wall layers within only 15 min, depending on specimen size and folding.

The novelty of this semi-automatic method is justified by the combination of detailed manual alignment, which has been developed within the program to allow highly precise adjustments, and the high accuracy of the automated calculations, which provide densely positioned measurement points. This enables the program to generate precise information on, in this case, the histomorphometry of the bowel which is novel information that has previously been unavailable. To the best of our knowledge, no comparable software for histomorphometry is currently available. Whereas the caliper tools in conventional histopathology image management software, such as Sectra PACS ID7 (Sectra AB, Linköping, Sweden) used at our institution, are designed primarily for the assessment of cell dimensions and resection margins, the program developed here provides the capability of more detailed and accurate measurements. Owing to the sparse sampling of measurement points, manual assessments are hypothetically expected to be less efficient and to approximate the true mean layer thickness with a possible lower accuracy than the semi-automatic method. The difference was shown in the agreement analyses between semi-automated and manual assessments, in which all layers diverged from good reliability and strong agreements.

The inter-observer reliability ranged from moderate to good, as reflected by the ICC values. The high levels of both reliability and agreement between observers, strongly suggested that histomorphometry will need only one observer’s assessment in future analysis, provided that robust image quality is maintained to ensure high data quality. In the inter-observer validation, a good reliability (ICC) was observed for all histoanatomic layers except for the myenteric layer (aganglionosis and ganglionosis) which showed poor reliability, and the muscularis externa (ganglionosis) and mucosa (aganglionosis) showing moderate reliability. The highest inter-observer agreements, as demonstrated in the Bland-Altman plots, was observed in the analyses of the muscularis interna and submucosa. In these plots, a small subset of individual comparisons exceeded the ± two standard deviation limits of agreement. Otherwise, the distribution appeared uniform, suggesting these observations may represent outliers. Confirmation of this interpretation would require a larger patient cohort. This observed consistency of significant muscularis interna results align with previous studies from our research group, regarding both histomorphometric analyses and analyses of ultrasound images [4, 6, 7, 9]. The finding underscores the muscularis interna as a pivotal layer for investigating histomorphometric features of Hirschsprung’s disease. The weakest inter-observer reliability and agreement was observed for the myenteric tissue layer, highlighting the challenges in identifying and measuring the presence and thickness of this layer specifically. The myenteric tissue layer demonstrated considerable variation in thickness both within individual images and across patients. In addition, observers differed in their assessment of whether the layer was present in certain images, indicating that this layer is particularly prone to interpretative variability. Moreover, due to the thinness of the layer, even minor delineation errors can substantially affect reliability and agreement metrics.

In our previously developed similar software for bowel wall assessment using ultra-high frequency ultrasound [9], the inter-observer validation demonstrated an overall higher inter-observer reliability compared with the present histopathology study. This difference could speculatively be attributed to the shorter delineation lines in the area of interest in ultrasound images, compared with the histopathology program, where measurements were taken on full circumferential cross-sections, potentially increasing variability.

The intra-observer reliability analyses were good to excellent, with the highest ICCs observed for the muscularis externa and interna indicating that repeated measurements by one observer do not seem to be necessary in future analyses, at least regarding the muscularis layers. However, as with the inter-observer analyses, the myenteric layer also showed the lowest reliability and agreement in intra-observer analyses, underscoring its inconsistency and uncertainty.

Comparing the computer program-generated and manually assessed histomorphometry, the results of reliability and agreement analyses varied largely between the various bowel wall layers, overall presenting in lower ranges. The overall weaker reliability between manual assessment and the computer program might stem from the software’s enhanced accuracy, as it calculates mean thicknesses based on a higher sampling density of thousands of individual point measurements. The rougher manual assessment allowed only 10 measurements per layer and may therefore be expected to be less representative of the true mean layer thickness. The reliability differed especially between the muscularis externa in aganglionic and ganglionic bowel and was best in ganglionic bowel. Speculatively, variability between the manual and semi-automatic measurements may relate to differences in the inclusion of the serosa into the muscularis externa, to the difficulty of consistently identifying the myenteric layer, and maybe also to fatigue-related variability associated with the time-consuming manual procedure.

A major strength of this development and validation study is the novelty of the in-house programmed computer software, and its development within a translational team with years of collaboration and experience. Continuous refinements were ensured through iterative discussions and feedback. Another strength was the rigorous validation of each histoanatomical layer using different methods, which increases the program’s generalizability, enabling potential applications to other gastrointestinal diseases, such as inflammatory bowel disease, and possibly to other organs. The program has previously been applied to histomorphometric analysis of bowel wall in patients with Hirschsprung’s disease, revealing systematic differences between aganglionic and ganglionic bowel, and demonstrating correlation between histopathology and ultra-high frequency ultrasound imaging [7, 15]. A key limitation of this study is that the computer assessments and manual measurements were performed at different timepoints, potentially leading to discrepancies in the definition of certain layers. This could be the case for discrepancies in agreement of the myenteric tissue layer and the muscularis externa in the semi-automatic versus manual comparison. Another limitation was that the full bowel wall thickness was derived from a composite of individual layer measurements, potentially skewing reliability and agreement. An important consideration when using the computer program is the prerequisite of the observer’s foundational histoanatomical knowledge to accurately assess and delineate specific bowel wall layers. In future studies, the computer program may be used to assess the transition zone between the aganglionic and ganglionic bowel wall. With respect to the reliable identification of histoanatomical layers in frozen-section biopsy, the program has not yet been applied in this context. This may be challenging, as histological staining probably will still be required to accurately delineate the layers in the images exported to the program. Although no primary clinical application has yet been demonstrated for this histopathology program, it is already used in the development of future precision diagnostics. It may also have relevance beyond bowel disorders, potentially offering histomorphometric insights in other organ systems.

## Conclusions

The computer program developed and validated for the assessment of bowel wall histoanatomic morphometrics represents an innovative software application that enables precise and efficient analysis of histoanatomic thicknesses. Overall, both inter- and intra-observer reliability and agreement are strong, particularly for measurements of the muscularis propria layers. These findings demonstrate accurate performance and position the program as a promising tool for advancing imaging-based diagnostic techniques rooted in histomorphometrics, including applications in Hirschsprung’s disease.

## Supplementary Information

Supplementary Material 1.

## Data Availability

The datasets used and analysed during the current study are available from the corresponding author on reasonable request.

## Abbreviations

ICC: Intraclass Correlation Coefficient
