# Supplementary material for: Development and validation of a computer program for histoanatomical morphometric analysis of the bowel wall in children with Hirschsprung’s disease
Source: Diagn Pathol. 2026 Mar 21;21:37. doi: 10.1186/s13000-026-01779-7 (PMC13063597; doi:10.1186/s13000-026-01779-7)
Supplement: Supplementary file 1 — Supplementary Material 1. [file 13000_2026_1779_MOESM1_ESM.docx]

# Supplementary materials

**Table 1.** **Inter-observer reliability of a computer program for histomorphometric analysis. Comparison of aganglionic segments.**

| **Histoanatomical layer** | **Thickness observer 1**  ***Median (Range)*** | **Thickness observer 2**  ***Median (Range)*** | **Absolute difference**  ***Median (Range)*** | **ICC^1^** |
| --- | --- | --- | --- | --- |
| **Myenteric tissue layer (mm)**  **n=37** | \| 0.021  (0.001 – 0.095) \| \| --- \| | \| 0.010  (0.002 – 0.081) \| \| --- \| \|  \| | \| 0.009  (-0.042 - 0.071) \| \| --- \| | 0.264 |
| **Submucosa (mm)**  **n=37** | \| 0.553  (0.141 – 1.155) \| \| --- \| \|  \| | \| 0.540  (0.193 – 1.455) \| \| --- \| \|  \| | \| -0.004  (-0.430 - 0.385) \| \| --- \| | 0.844 |
| **Mucosa (mm)**  **n=37** | \| 0.429  (0.204 – 1.324) \| \| --- \| \|  \| | \| 0.499  (0.190 – 1.335) \| \| --- \| \|  \| | \| -0.026  (-0.741 - 0.585) \| \| --- \| \|  \| | 0.577 |
| **Full bowel wall (mm) n=37** | 1.703  (0.936 – 3.662) | \| 1.688  (1.120 – 3.501) \| \| --- \| | 0.011  (-1.619 - 1.048) | 0.829 |

^1^ Intraclass Correlation Coefficient (ICC) <0.5 poor reliability; ICC 0.5 - 0.75 moderate reliability; ICC 0.75 - 0.9 good reliability; ICC>0.9 excellent reliability.

The table presents inter-observer reliability between two observers using the in-house developed computer program for assessment of bowel wall histomorphometry in histopathology images of resected bowel specimens in children with Hirschsprung’s disease.

**Table 2.** **Inter-observer reliability of a computer program for histomorphometric analysis. Comparison of ganglionic segments.**

| **Histoanatomical layer** | **Thickness observer 1**  ***Median (Range)*** | **Thickness observer 2**  ***Median (Range)*** | **Absolute difference**  ***Median (Range)*** | **ICC^1^** |
| --- | --- | --- | --- | --- |
| **Myenteric tissue layer (mm)**  **n=38** | \| 0.028  (0.001 - 0.175) \| \| --- \| \|  \| | \| 0.018  (0.002 - 0.068) \| \| --- \| \|  \| | \| 0.009  (-0.051 - 0.138) \| \| --- \| \|  \| | 0.101 |
| **Submucosa (mm)**  **n=38** | \| 0.486  (0.226 - 1.140) \| \| --- \| \|  \| | \| 0.462  (0.235 - 1.340) \| \| --- \| \|  \| | \| -0.001  (-0.619 - 0.243) \| \| --- \| \|  \| | 0.882 |
| **Mucosa (mm)**  **n=38** | \| 0.433  (-3,436 - 1.000) \| \| --- \| | \| 0.433  (0.178 - 0.875) \| \| --- \| | \| 0.451  (0.178 - 0.929) \| \| --- \| | 0.836 |
| **Full bowel wall (mm) n=38** | 1.898  (1.061 - 3,384) | 1.897  (1.071 - 4,007) | 0.035  (-1.808 - 1.064) | 0.818 |

^1^ Intraclass Correlation Coefficient (ICC) <0.5 poor reliability; ICC 0.5 - 0.75 moderate reliability; ICC 0.75 - 0.9 good reliability; ICC>0.9 excellent reliability.

The table presents inter-observer reliability and agreement between two observers using the in-house developed computer program for assessment of bowel wall histomorphometry in histopathology images of resected bowel specimens in children with Hirschsprung’s disease.

**Table 3.** **Intra-observer reliability of a computer program for histomorphometric analysis. Comparison of aganglionic segments**

| **Histoanatomic Layers** | **Thickness first measurement**  ***Median (Range)*** | **Thickness second measurement**  ***Median (Range)*** | **Absolute difference**  ***Median (Range)*** | **ICC^1^** |
| --- | --- | --- | --- | --- |
| **Muscularis externa (mm)**  **n=37** | \| 0.316  (0.164 - 1.227) \| \| --- \| \|  \| | \| 0.286  (0.146 - 1.221) \| \| --- \| \|  \| | 0.011  (-0.080 - 0.209) | 0.969 |
| **Muscularis interna (mm)**  **n=37** | \| 0.419  (0.235 - 0.752) \| \| --- \| \|  \| | \| 0.379  (0.249 - 0.835) \| \| --- \| \|  \| | -0.001  (-0.132 - 0.080) | 0.892 |
| **Myenteric tissue layer (mm)**  **n=37** | \| 0.021  (0.000 - 0.095) \| \| --- \| \|  \| | \| 0.021  (0.003 - 0.089) \| \| --- \| \|  \| | -0.002  (-0.033 - 0.043)   \|  \| \| --- \| | 0.757 |
| **Submucosa (mm)**  **n=37** | \| 0.553  (0.141 - 1.155) \| \| --- \| \|  \| | \| 0.561  (0.205 - 1.212) \| \| --- \| \|  \| | 0.000  (-0.172 - 0.393)   \|  \| \| --- \| | 0.932 |
| **Mucosa (mm)**  **n=37** | \| 0.429  (0.204 - 1.324) \| \| --- \| | 0.468  (0.201 - 0.647) | 0.000  (-0.117 - 0.696) | 0.698 |
| **Full bowel wall (mm) n=37** | \| 1.703  (0.936 - 3,662) \| \| --- \| | \| 1.727  (0.910 - 3,563) \| \| --- \| | -0.006  (-0.243 - 1.052) | 0.935 |

^1^ Intraclass Correlation Coefficient (ICC) <0.5 poor reliability; ICC 0.5 - 0.75 moderate reliability; ICC 0.75 - 0.9 good reliability; ICC>0.9 excellent reliability.

The table presents intra-observer reliability between one observer’s measurements and re-measurements using the in-house developed computer program for assessment of bowel wall histomorphometry in histopathology images of resected bowel specimens in children with Hirschsprung’s disease.

**Table 4. Intra-observer reliability of a computer program for histomorphometric analysis. Comparison of ganglionic segments.**

| **Histoanatomical layers** | **Thickness first measurement**  ***Median (Range)*** | **Thickness second measurement**  ***Median (Range)*** | **Absolute difference**  ***Median (Range)*** | **ICC**^1^ |
| --- | --- | --- | --- | --- |
| **Muscularis externa (mm)**  **n=38** | \| 0.326  (0.130 - 0.679) \| \| --- \| \|  \| | \| 0.299  (0.122 - 0.623) \| \| --- \| \|  \| | \| 0.009  (-0.093 - 0.132) \| \| --- \| \|  \| | 0.921 |
| **Muscularis interna (mm)**  **n=38** | \| 0.620  (0.204 - 0.994) \| \| --- \| \|  \| | \| 0.614  (0.228 - 1.025) \| \| --- \| \|  \| | \| -0.009  (-0.135 - 0.067) \| \| --- \| | 0.972 |
| **Myenteric tissue layer (mm)**  **n=38** | \| 0.026  (0.000 - 0.175) \| \| --- \| \|  \| | \| 0.031  (0.003 - 0.073) \| \| --- \| \|  \| | \| 0.005  (-0.055 - 0.109) \| \| --- \| \|  \| | 0.418 |
| **Submucosa (mm)**  **n=38** | \| 0.486  (0.226 - 1.140) \| \| --- \| \|  \| | \| 0.472  (0.231 - 1.123) \| \| --- \| \|  \| | \| -0.001  (-0.147 - 0.074) \| \| --- \| \|  \| | 0.983 |
| **Mucosa (mm)**  **n=38** | 0.433  (0.178 - 0.875) | 0.422  (0.175 - 0.873) | 0.003  (-0.082 - 0.160) | 0.971 |
| **Full bowel wall (mm) n=38** | 1.898  (1.061 - 3,384) | 1.887  (1.039 - 3,358) | 0.018  (-0.386 - 0.180) | 0.987 |

^1^ Intraclass Correlation Coefficient (ICC) <0.5 poor reliability; ICC 0.5 - 0.75 moderate reliability; ICC 0.75 - 0.9 good reliability; ICC>0.9 excellent reliability.

The table presents intra-observer reliability between one observer’s measurements and re-measurements using the in-house developed computer program for assessment of bowel wall histomorphometry in histopathology images of resected bowel specimens in children with Hirschsprung’s disease.

**Table 5.** **Reliability between manual measurements and a computer program for** **histomorphometric analysis. Comparison of aganglionic segments.**

| **Histoanatomical**  **layers** | **Thickness computer program measurements**  ***Median (Range)*** | **Thickness manual measurements**  ***Median (Range)*** | **Absolute difference**  ***Median (Range)*** | **ICC^1^** |
| --- | --- | --- | --- | --- |
| **Myenteric tissue layer (mm)**  **n=17** | 0.031  (0.002 - 0.095) | 0.029  (0.006 - 0.143) | 0.001  (-0.100 - 0.053) | 0.164 |
| **Submucosa (mm)**  **n=17** | 0.585  (0.141 - 1.155) | 0.353  (0.158 - 1.156) | 0.175  (-0.064 - 0.562) | 0.636 |
| **Mucosa (mm)**  **n=17** | 0.419  (0.238 - 1.324) | 0.519  (0.356 - 0.702) | -0.111  (-0.221 - 0.689) | 0.385 |

^1^ Intraclass Correlation Coefficient (ICC) <0.5 poor reliability; ICC 0.5 - 0.75 moderate reliability; ICC 0.75 - 0.9 good reliability; ICC>0.9 excellent reliability.

The table presents reliability between manual measurements and the in-house developed computer program for assessment of bowel wall histomorphometry in histopathology images of resected bowel specimens in children with Hirschsprung’s disease.

**Table 6. Reliability between manual measurements and a computer program for** **histomorphometric analysis. Comparison of ganglionic segments.**

| **Histoanatomical**  **layers** | **Thickness computer program measurements**  ***Median (Range)*** | **Thickness manual measurements**  ***Median (Range)*** | **Absolute difference**  ***Median (Range)*** | **ICC^1^** |
| --- | --- | --- | --- | --- |
| **Myenteric tissue layer (mm)**  **n=18** | 0.030  (0.007 - 0.089) | 0.029  (0.015 - 0.046) | -0.006  (-0.029 - 0.063) | -0.166 |
| **Submucosa (mm)**  **n=18** | 0.512  (0.277 - 1.048) | 0.298  (0.178 - 0.846) | 0.187  (0.072 - 0.447) | 0.547 |
| **Mucosa (mm)**  **n=18** | 0.478  (0.329 - 0.875) | 0.510  (0.379 - 1.038) | -0.049  (-0.216 - 0.281) | 0.777 |

^1^ Intraclass Correlation Coefficient (ICC) <0.5 poor reliability; ICC 0.5 - 0.75 moderate reliability; ICC 0.75 - 0.9 good reliability; ICC>0.9 excellent reliability.

The table presents reliability between manual measurements and the in-house developed computer program for assessment of bowel wall histomorphometry in histopathology images of resected bowel specimens in children with Hirschsprung’s disease.





**Figure 1. Bland-Altman plots visualizing inter-observer agreement of layer thickness measurements of the bowel wall.**

**Figure 1 legend:** Inter-observer agreement between two observers assessing bowel layer thickness measurements from histopathological images analyzed in the in-house developed computer program for automatic histomorphometric bowel wall assessment. The images were of aganglionic and ganglionic bowel wall from children operated on for Hirschsprung’s disease. The blue horizontal lines represent the mean thickness difference. The yellow dashed horizontal lines indicate the limits of agreement, defined as the mean thickness difference ± two standard deviations of the differences.

**

**

**Figure 2. Bland-Altman plots visualizing intra-observer agreement of layer thickness measurements of the bowel wall.**

**Figure 2 legend:** Inter-observer agreement of one observer’s measurements and re-measurements assessing bowel layer thickness measurements from histopathological images analyzed in the in-house developed computer program for automatic histomorphometric bowel wall assessment. The images were of aganglionic and ganglionic bowel wall from children operated on for Hirschsprung’s disease. The blue horizontal lines represent the mean thickness difference. The yellow dashed horizontal lines indicate the limits of agreement, defined as the mean thickness difference ± two standard deviations of the differences.

**

**

**Figure 3. Bland-Altman plots visualizing agreement between manual and computer program measurements of bowel wall layer thicknesses.**

**Figure 3 legend.** Agreement between the in-house developed computer program for automatic histomorphometric bowel wall assessment and manual assessment of layer thickness measurements from histopathological images. The images were of aganglionic and ganglionic bowel wall from children operated on for Hirschsprung’s disease. The blue horizontal lines represent the mean thickness difference. The yellow dashed horizontal lines indicate the limits of agreement, defined as the mean thickness difference ± two standard deviations of the differences.
